# Women’s support for voluntary medical male circumcision in fishing communities on the shores of Lake Victoria, Uganda

**DOI:** 10.1186/s12913-022-07842-5

**Published:** 2022-04-14

**Authors:** Aggrey Byaruhanga, Nazarius Tumwesigye Mbona, Suzan Babirye, Fred Nalugoda, Edward Nelson Kankaka, Lucas Ampaire, Richard Migisha, Joseph Kagaayi

**Affiliations:** 1 Makerere University School of Public Health, P.O Box 7072, Kampala, Uganda; 2 Rakai Health Science Program, Rakai, Uganda; 3 Mbarara University of Science and Technology, Mbarara, Uganda

**Keywords:** Voluntary Medical Male Circumcision, HIV, Fishing communities, Uganda

## Abstract

**Background:**

Women’s support can improve uptake of voluntary medical male circumcision (VMMC). We assessed the level of women’s support for VMMC and associated factors in fishing settlements on the shores of Lake Victoria in Uganda, to inform interventions aimed at increasing the uptake of safe male circumcision services in such high-risk populations.

**Methods:**

We conducted a cross-sectional study, employing mixed methods of data collection, at Kasenyi and Kigungu landing sites in April 2018. We included women aged 18–49 years, who had stayed at the landing sites for ≥3 months. We obtained qualitative data using focus group discussions (FGDs), and interviewer-administered semi-structured questionnaires for quantitative data. The tool captured demographic characteristics, community factors including cultural norms and beliefs, women’s experiences, and health facility-related factors. The dependent variable was derived from the response to the question: "Would you encourage your partner/husband to go for VMMC?", and used as a proxy for support of VMMC. We used modified Poisson regression to identify factors associated with women’s support for VMMC. Qualitative data were analysed using thematic content analysis.

**Results:**

We enrolled 313 women with a mean age of 28 (SD±6.8) years. Of the 313 women, 230 (73.5%) supported VMMC. Belief that VMMC increases penile hygiene (Adjusted prevalence ratio [aPR]=1.9; CI: 1.8–3.2), performing VMMC for religious reasons (aPR=1.9; CI: 1.8–2.9), preference for a circumcised man (aPR=1.3; CI: 1.2–1.5), belief that vaginal fluids facilitate wound healing (aPR=1.9; CI: 1.3–2.7), and knowledge about when a man can resume sex (4 weeks) after circumcision (aPR=2.1; CI: 1.8–3.3) were associated with women’s support for VMMC. FGDs revealed that women were not adequately involved in VMMC activities for decision making.

**Conclusion:**

The support for VMMC was high among women in the fishing communities. However, women perceived they were not involved in decision-making for VMMC and had several misconceptions, including a belief that vaginal fluids facilitate wound healing. The Ministry of Health and VMMC implementing partners should devise strategies to increase sensitization and involvement of women in VMMC decision-making without slowing service uptake.

## Introduction

Voluntary medical male circumcision (VMMC) is a surgical procedure that involves the removal of the foreskin by a trained medical professional is effective in the prevention of HIV transmission [1]. Apart from HIV prevention, VMMC benefits women through improving hygiene, lowering the risk for sexually transmitted infections (STIs), and reducing the risk of Human Papilloma Virus (HPV)-associated cervical cancer [1, 2]. Men’s health is as much about women’s health when it comes to sexually transmitted diseases [3]. Three randomized controlled trials confirmed that male circumcision reduces female to male HIV transmission by approximately 60% [4–6]. On the basis of these findings, the World Health Organization (WHO) and the Joint United Nations Programme on HIV/AIDS (UNAIDS) recommended the inclusion of voluntary medical male circumcision (VMMC) into HIV prevention programs for countries with high HIV prevalence and low male circumcision rates. Furthermore, epidemiological and economic modeling of the impact and cost of medical male circumcision (MMC) scale-up showed that VMMC was cost-effective [7]. Uganda was identified in the Eastern and Southern African region as a priority country for VMMC scale-up; however, it had only achieved 45 % of its target by June 2020 [8].

A prospective community cohort study conducted from 2011 to 2017 in four fishing communities on the shores of Lake Victoria, Uganda, reported an increased coverage of safe male circumcision from 35% to 65%; however, the same study observed that the risky sexual behaviors did not decrease over the 5 years [9]. Moreover, the coverage for VMMC among non-Muslim youths in some of the fishing communities was reported to be at 54%, way below the national target of 80% [10].

Although men are the main focus of VMMC services, literature shows that women can play a key role in the scaling up of VMMC [11–13]. Women’s beliefs about VMMC and their endorsement significantly influence their male partners [14–16]. A community randomized trial in Uganda found that men were more likely to undergo VMMC in the intervention arm due to their female partners’ influence [17]. Women may therefore be valuable in strategies for increasing VMMC services uptake [17]. A study conducted in Zambia also reported that discussing VMMC with a female sexual partner was the greatest predictor of readiness to undergo VMMC by their male counterpart [15].

Fisherfolks are among key populations with high HIV prevalence ranging from 14 to 40% [9, 18, 19]. Fishing communities have unique risk factors that render persons in these settlements vulnerable to high rates of HIV infections. These include the mobility and migratory nature of the fishing industry [20], absence of social structures, high rates of alcohol consumption, transactional sex, concurrent sexual partnerships, low rates of condom use, and inadequate knowledge about HIV transmission [21–24]. In addition, prevention interventions in these communities, including safe male circumcision may not be readily accessible [25, 26].

Women in fishing communities need to participate in HIV prevention [27]. Women are an important audience for VMMC communication messages so that they know that VMMC provides some, but only partial protection against HIV [11]. However, the level at which women are engaged in VMMC scale-up in major fishing settlements in Uganda, including Kasenyi and Kigungu landing sites or fishing communities around Lake Victoria, is unknown. Studies conducted previously in fishing communities have qualitatively focused on perceptions of women towards VMMC [28], but not the level of support. We established the level of, and factors associated with women’s support for VMMC in Kasenyi and Kigungu landing sites to inform interventions aimed at increasing the uptake of VMMC services in such high-risk populations.

## Methods

### Study setting

**Fig. 1:**
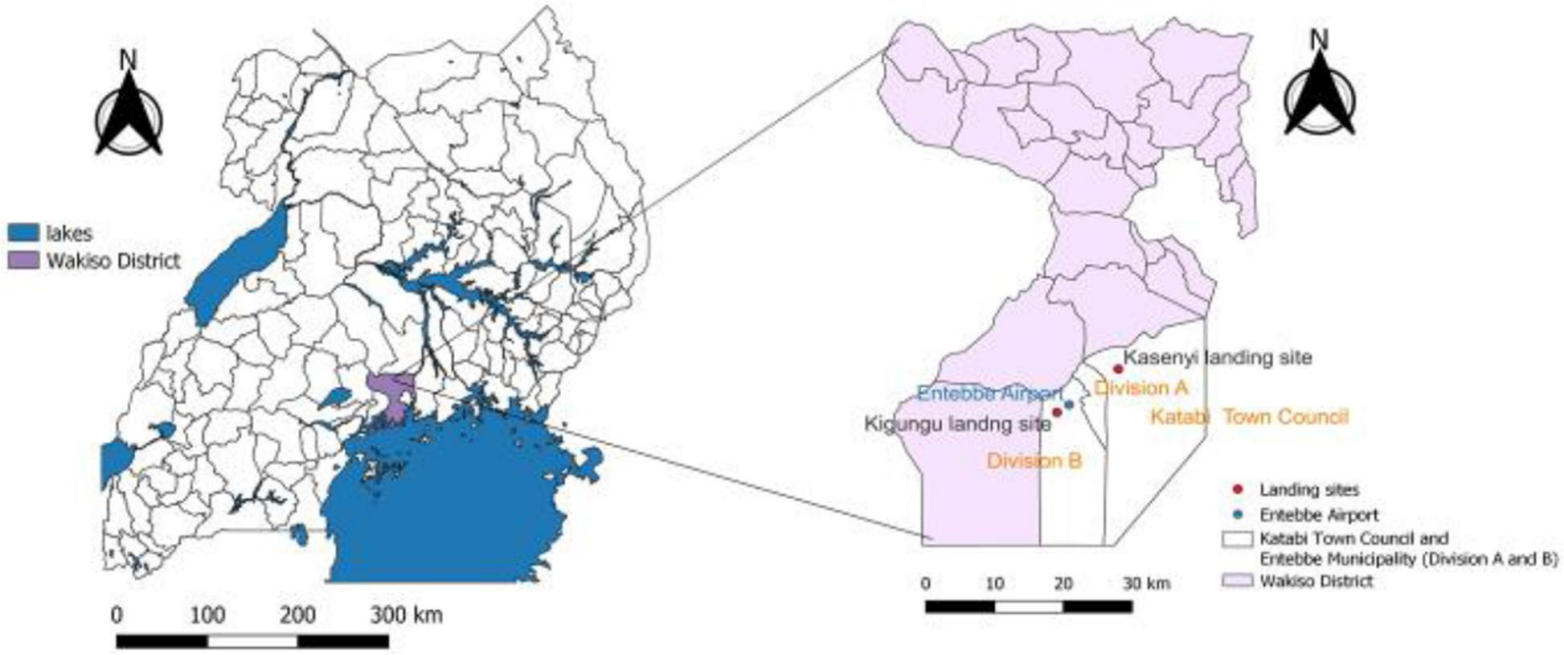
A map showing the location of Kasenyi and Kigungu landing sites on the shores of Lake Victoria, Wakiso District, Uganda

The study was conducted in Kasenyi and Kigungu landing sites on the shores of Lake Victoria from April 12-28, 2018. Kasenyi landing site is among the most significant landing site located along the northern part of Lake Victoria shoreline and is found in Nkumba Parish, Katabi Sub-county, while Kigungu landing site is found in Kigungu Parish bordering Entebbe international Airport, in Entebbe Municipality, Wakiso District (Fig. 1). The two landing sites are highly overpopulated and are 50 km away from Kampala, the country’s capital city, and about 20 km apart from each other. The landing sites are known to have a high burden of HIV, with reported HIV prevalence estimates ranging from 25.5% to 40% [24, 29–31].

### Study population and inclusion criteria

We included women aged 18-49 years who had stayed at the two landing sites for at least three months, and who were directly or indirectly involved in fishing work or work supporting the fishing industry. We included women in the age group of 18-49 years who are legally married women and who are premenopausal. We excluded women who declined to consent to the study.

### Study design

This was a cross-sectional study that employed both quantitative and qualitative methods of data collection.

### Sample size determination

We determined the sample size using Kish Leslie (1965) formula [32] with the following assumptions: the estimated proportion of women supporting VMMC 50%, the standard normal value α at 95% level of significance, and a maximum error at 5 %. We assumed a 50% level of support because no previous study had been conducted in fishing communities to determine the level of women’s support for VMMC) [33]. Considering a non-response rate of 10%, we estimated a total sample size of 428 women.

### Sampling procedures

For the quantitative data, we used systematic sampling. This was done by obtaining the total number of households in the two landing sites from which the sampling interval, n^th^ was calculated below;

$${\mathrm{n}}^{\mathrm{th}}=\frac{\mathrm{Total}\ \mathrm{number}\ \mathrm{of}\ \mathrm{households}\ \mathrm{in}\ \mathrm{the}\ \mathrm{landing}\ \mathrm{site}}{\mathrm{The}\ \mathrm{sample}\ \mathrm{size}\ \mathrm{of}\ \mathrm{households}\ \mathrm{needed}\ \mathrm{in}\ \mathrm{that}\ \mathrm{landing}\ \mathrm{site}}$$

$$\mathrm{For}\ \mathrm{Kasenyi},\kern0.5em {\mathrm{n}}^{\mathrm{th}}=\frac{1200}{321}=4,\kern0.5em \mathrm{every}\ \mathrm{fourth}\ \mathrm{household}\ \mathrm{was}\ \mathrm{selected}$$

$$\mathrm{Kigungu},\kern0.5em {\mathrm{n}}^{\mathrm{th}}=\frac{600}{107},=6,\kern0.5em \mathrm{every}\ \mathrm{sixth}\ \mathrm{household}\ \mathrm{was}\ \mathrm{selected}.$$

A list of households was obtained from the chairpersons of the two landing sites. The edge of the landing site was identified with the help of the chairperson; we stood and rolled a pen to determine the direction to take. The first house in that direction was the starting point; after which a linear transect walk was done sampling every ‘n^th^’ house in that direction. After finishing the edge of the landing site before completing the required sample, we took another random direction using the rolling pen again. With the household having more than one eligible participant, we randomly selected the participant from that household. Papers were handwritten with “YES and NO”, any participant who picked yes was interviewed.

For qualitative data, we conducted four focus group discussions (FGDs), with a total of 32 participants. Women selected were stratified by age (18-30 years, and 31-49 years). A total of 8-12 participants were invited for each discussion. We mobilized the women in convenient places (in the compounds of the Local Council chairpersons) for the meetings. Before conducting the FGD, the place and time for the meeting were agreed upon with the participants. Consent for the recording was sought from each FGD participant. All participants were given the chance to contribute to the discussion. Each interview took approximately one hour.

### Study tools

For the quantitative data, we interviewed the women one on one using a standardized semi-structured questionnaire by trained research assistants, who were health workers (nurses and clinical officers). A team of 4 researchers participated in the design and review of the questionnaire. We trained the health workers on data collection procedures. The selected health workers had prior knowledge about VMMC and were able to speak both English and the local language (Luganda). The questionnaire was pre-tested at Nakiwogo landing site found on the shores of Lake Victoria to remove ambiguous questions. For qualitative data, we used an FGD guide.

### Study variables

The dependent variable was women’s support for Voluntary medical male circumcision. The dependent variable was a dichotomous indicator variable derived from the answer given to the following question: “Would you encourage your partner/ husband to go for voluntary medical male circumcision?” This variable was coded yes=1 or no=0 and used as a proxy for support of VMMC.

The independent study variables were selected based on the review of existing literature. Individual characteristics of respondents included age, marital status, level of education, religion, place of residence, and occupation. The tool also captured knowledge on the role of VMMC in the prevention of HIV/AIDS. Community-related factors included misconceptions about VMMC, such as fear of increased promiscuity among circumcised men or the misconception that VMMC provides complete protection against HIV; cultural beliefs that married men should have sex with virgin girls after circumcision to promote healing, VMMC done for cultural and religious reasons, foreskins are sold after circumcision, vaginal fluids facilitate wound healing, and whether VMMC improves penile hygiene were captured. Health facility-related factors that were assessed included availability of information education communication materials on VMMC, negative attitudes of health workers, the cost of VMMC services, and inadequacy of health workers in health facilities. We explored women’s experiences about VMMC, including involving them in VMMC decision-making by their partners, preference of having sex with circumcised men, and abstaining from sex before the healing period of their circumcised partners.

### Data management and analysis

For quantitative data, we checked the questionnaires for completeness and accuracy of the responses. We entered the data into the EPI DATA version 3.02 (*EpiData,* Odense, Denmark) and exported it to STATA version 14.0 (*StataCorp*, College Station, Texas, USA) for analysis. Descriptive statistics were presented as means (standard deviation) and medians (interquartile range) for continuous variables (e.g, age); and frequencies and proportions for categorical variables.

The level of women’s support for VMMC was determined as a proportion of participants who responded that they would encourage their partners to go for VMMC, expressed as a percentage. To identify the factors associated with women’s support for VMMC, we used modified Poisson regression. Variables that were significant at bivariable analysis were checked for collinearity and included in the multivariable model to estimate independent effects. Variables in the final model were considered statistically significant at a p-value <0.05

For qualitative data, we audio-recorded the FGDs, made notes, and transcribed them from the local language (Luganda) to the English version. Transcripts were read several times with the help of research assistants to get a general impression of the data. Repeated issues were developed into categories and further grouped into themes. We analyzed qualitative data using ATLAS ti.8 and summarized it according to themes. We presented the main results verbatim.

## Results

We interviewed 313 women from the estimated sample size of 428 (the non-response rate was 26.9%). This was due to the mobility of women in fishing communities; most of them were not found in their households.

The respondents were in the range of 18-49 years with a mean of 28 (S.D±6.8) years. Most of the women were in the age bracket of 18-29 years (64.2%), and approximately one-half (51.4%) had attained secondary education (Table 1).

**Table 1:** Socio-demographic characteristics of women in fishing communities along the shores of Lake Victoria, Uganda

Variables	Total (%ge)	Support of VMMC		x^**2**^	***p***-value
	***n***=313	Yes(***n***=230)	No(***n***=83)		
**Age in years**					
Mean (S.D) 27.8 (6.8)					
Median (IQR) 27 (22-32)					
**Age in years**					
18-29	201 (64.2)	168 (64.9)	32 (60.4)	4.1	0.04
30-39	93 (29.7)	79 (30.5)	14 (26.4)		
40-49	19 (6.1)	12 (4.6)	07 (13.2)		
**Level of Education**					
No formal education	21 (6.8)	16 (6.4)	05 (7.9)	2.3	0.09
Primary	105 (33.5)	81 (32.4)	24 (38.1)		
Secondary	161 (51.4)	138 (55.2)	23 (36.5)		
Tertiary	26 (8.3)	15 (6.0)	11 (17.5)		
**Religion**					
Roman Catholic	123 (39.5)	107 (41.2)	16 (30.2)	7.5	0.01
Protestan**t**	89 (28.4)	70 (26.9)	19 (35.8)		
Muslim	49 (15.6)	36 (13.8)	13 (24.6)		
Others	52 (16.5)	47 (18.1)	05 (9.4)		
**Current relation status**					
Single	104 (36.4)	84 (33.7)	20 (31.7)	3.9	0.05
Married	123 (39.6)	105 (42)	18 (28.6)		
Divorced	60 (19.2)	45 (18)	15 (23.8)		
Widowed	26 (5.1)	16 (6.4)	10 (15.9)		
**Occupation**					
Bar Attendant	27 (8.7)	20 (7.7)	07 (13.5)	1.9	0.7
Restaurant Attendant	93 (29.8)	76 (29.2)	17 (32.7)		
Trader	147 (47.1)	129 (49.6)	18 (34.6)		
Others	36 (14.4)	35 (13.4)	10 (19.2)		

### The level of voluntary medical male circumcision support, and associated factors among women living on the shores of Lake Victoria, Uganda

Of the 313 women interviewed, 230 (73.5%, 95% CI: 68.2-78.3) responded that they would encourage their partners to go for voluntary medical male circumcision.

At multivariable analysis (Table 2), the prevalence of support for VMMC was 1.3 times higher among women who preferred to have sexual intercourse with circumcised men after adjusting for age, tribe, and religion (aPR= 1.3, 95% CI: 1.2-1.5).

**Table 2:** Adjusted prevalence ratios of women’s support for VMMC for their partners, in fishing communities on the shores of Lake Victoria, Uganda

Variable	Support for VMMC		cPR (95%CI)	aPR (95%CI)
	Yes (%ge)	No (%ge)		
**Age in years**				
18-29	168 (64.9)	32 (60.4)	REF	REF
30-39	79 (30.5)	14 (26.4)	0.99 (0.92-1.1)	1.0 (0.95-1.6)
40-49	12 (4.6)	07 (13.2)	0.18 (0.01-0.4)*	1.14 (0.9-1.3)
**Religion**				
Catholics	107 (41.2)	16 (24.2)	REF	REF
Protestants	70 (26.9)	19 (28.7)	1.08 (0.9-1.2)	1.0 (0.9-1.1)
Muslims	36 (13.8)	13 (39.4)	1.3 (1.0-1.5)**	1.06 (0.9-1.2)
Others	47 (18.1)	05 (7.7)	0.97 (0.8-1.0)	1.32 (0.9-1.8)
**Prefer a circumcised man**				
No	07 (2.7)	25 (34.9)	REF	REF
Yes	246 (89.8)	28 (65.1)	1.5 (1.4-1.7)***	1.3 (1.2-1.5)***
**Resumption of sex after male circumcision**				
2 weeks	52 (21.6)	13 (20.6)	REF	REF
4 weeks	126 (52.5)	37 (58.8)	1.8 (1.7-1.9)**	2.1 (1.8-3.3)**
6 weeks	62 (25.8)	13 (20.6)	1.02 (0.9-1.2)	1.01 (0.9-1.1)
**VMMC done for religious reasons**				
No	167 (64.2)	25 (47.2)	REF	REF
Yes	93 (35.8)	28 (52.8)	1.9 (1.8-2.1)**	1.9 (1.8-2.9)**
**Vaginal fluids heal wounds faster after circumcision**				
No	159 (61.2)	12 (22.6)	REF	REF
Yes	101 (38.8)	41 (77.4)	1.8 (1.7-2.9)***	1.9 (1.3-2.7)***
**VMMC increases penile hygiene**				
No	38 (14.6)	34 (47.2)	REF	REF
Yes	22 (85.4)	19 (7.9)	1.4 (1.3-1.5)***	1.2 (1.4-3.2)**

Significance *=*p*-value<0.05, **=*p*-value<0.01, ***=*p*-value<0.001

*REF* Reference category, *CI* Confidence interval, *cPR* Crude prevalence ratio, *aPR* Adjusted prevalence ratio, *VMMC* Voluntary medical male circumcision

Similarly, the prevalence of support for VMMC was 1.9 times higher among women who reported that VMMC is done for religious reasons compared to those who did not (aPR=1.9, 95% CI: 1.8-2.9). The prevalence of support for VMMC was 1.9 times higher among women who had a belief that vaginal fluids facilitate wound healing compared to those who did not (aPR=1.9, 95% CI: 1.3-2.7). Finally, the prevalence of support was 2.1 times higher among women who reported that their partners should resume sex after 4 weeks (aPR=2.1, 95% CI: 1.8-3.3) compared to those who reported two weeks.

### Experiences of women about voluntary medical male circumcision in Kasenyi and Kigungu Landing sites on the shores of Lake Victoria, Uganda

Women from these two fishing communities highlighted several critical issues that influence VMMC uptake by their husbands/partners. The results of this study are presented under two main themes that emerged from the FGDs: (1) benefits of VMMC and (2) barriers to VMMC.

#### Benefits of voluntary medical male circumcision

Improved penile hygiene was cited as a significant motivator to women’s support for VMMC. Many participants expressed a great pleasure to have sexual intercourse with circumcised men. They believed that circumcised men are always clean and offer greater sexual satisfaction compared to uncircumcised ones and are unable to infect their female partners with sexually transmitted infections (STIs).

*“I find that my boyfriend is not circumcised, for heavenly sake, I will tell him to go for circumcision, and if he refuses I run away; because he can infect me with sexually transmitted infections such candidiasis. This is because such infections (STIs) hide under the foreskin. But also, a circumcised man has a clean penis and one can easily enjoy sex with him.” (An FGD woman below 30 years from Kasenyi Landing site)*

### Barriers to Voluntary medical male circumcision support

#### Women not involved in voluntary medical male circumcision activities

Not adequately involving women in decision-making for VMMC programs was also cited as a barrier to VMMC support. All participants reported that they have never been involved in any VMMC activity. Health workers occasionally come to these fishing communities and invite only men to go for circumcision during outreaches.

*“Every time health workers come to this community, they invite only men to attend VMMC services. We are never involved in VMMC activities. These health workers may be, are not aware that we can show care to our husbands/partners by convincing them to go for circumcision.” (An FGD woman above 30 years from Kasenyi Landing site)*

#### Women taking care of the families during the wound healing period

The cost of taking care of families incurred by women during the wound healing period of their partners/ husbands was also cited as a barrier to VMMC support. Although most participants agreed that they would encourage their husbands/partners to go for VMMC, some of them reported that they have faced a lot of challenges taking care of their families when their husbands/partners were circumcised.

*“When my husband got circumcised, he spent more than two weeks at home without working. He was unable to go fishing since the wound had not healed. I was the one taking care of the family looking for money for school fees for our children and food to eat.” (An FGD woman above 30 years from Kigungu landing site)*

#### Concerns about the wound healing period

Participants were concerned about the wound healing period. They reported that circumcised men take a long time to heal (about 10 weeks). The perceived long healing period discourages men go for circumcision. Participants suggested that young men who do not have families should be encouraged to get circumcised.

*“Most men in this fishing community are not circumcised because of fear to leave their families starving. I was told that men who get circumcised take long to heal, maybe that’s why they normally refuse to go for circumcision. These young boys can manage to go for circumcision since they do not have families to take care of. But also, if the government can give little money to cater for the family during the healing period such incentives can motivate men to go for VMMC.” (An FGD woman above 30 years from Kigungu Landing site)*

## Discussion

This community-based cross-sectional study determined the level of women’s support for voluntary medical male circumcision for HIV prevention and associated factors in fishing communities on the shores of Lake Victoria, Uganda. The results show that nearly three-quarters (74%) of women were in support of their partners’ going for VMMC. Although the level of women’s support was high, they had several misconceptions about VMMC, including beliefs that VMMC is done for religious reasons, and that vaginal fluids facilitate wound healing. The study findings further reveal that a significant proportion of women lacked adequate knowledge about the abstinence period after VMMC, and perceived that they were not involved in VMMC activities.

The level of support for VMMC in the current study was high compared to previous studies conducted in Uganda and Zimbabwe, where 67% and 58% of women had support for VMMC respectively [17, 34]. This may be because women in the fishing communities —because of their vulnerabilities—have been targeted for other HIV prevention services, including HIV counselling and testing [27]. However, whether the high level of women’s support observed in our study translates into high VMMC rates in the fishing communities, should be explored in future studies.

In the current study, women acknowledged the benefits of VMMC, including improved penile hygiene, reduced risk of HIV transmission and other sexually transmitted infections, and increased sexual satisfaction. These findings are consistent with studies from Papua New Guinea and Zimbabwe [35, 36]. However, a significant proportion of the women in this study did not have adequate knowledge of the six weeks of wound healing after VMMC. This is in agreement with findings from a study conducted in Botswana [37]. Such inadequate knowledge may facilitate early resumption of sex [37, 38], thus creating an opportunity for increased risk of transmission of HIV infection. Furthermore, several misconceptions were significantly associated with women’s support for VMMC in the current study, including a belief that vaginal fluids heal the wound faster after circumcision. A previous study done in a nearby island district of Kalangala reported similar misconceptions [28]. Such misconceptions may negatively impact the scale-up of VMMC services in these fishing communities. On the basis of these findings, there is a need for ongoing awareness-raising campaigns in the fishing communities to dispel these myths and misconceptions.

Consistent with other studies done in East Africa, including Tanzania and Kenya [28, 39, 40], loss of time and income was identified as a barrier to VMMC uptake. This may be because many men in fishing communities rely on daily fishing income to support their families. In addition, fisher folks may require a longer healing period due to the nature of their jobs. For instance, during fishing activities, the circumcised penis may come into contact with unsanitary lake water. In fishing communities and other similar settings with a high HIV burden, VMMC-implementing partners may consider offering financial incentives or compensations to offset the economic costs for the lost time. Such financial incentives have proved to increase VMMC elsewhere [41].

In agreement with a previous study conducted in Kenya [42], women perceived they were not involved in VMMC services. Lack of women’s involvement in VMMC programs may demotivate them from encouraging their partners to undergo medical circumcision [11]. Thus, gender integration in VMMC may play a major role in the uptake of VMMC services and promote men's compliance to the six-week abstinence period during wound healing. Moreover, adverse outcomes may occur as a consequence of female partners' lack of involvement and knowledge.

Overall our study findings highlight the need to involve female partners in decision-making, to improve the uptake of VMMC services. Furthermore, the gender integration in VMMC will also empower women with adequate knowledge thereby dispelling the myths and misconceptions about VMMC. To optimize the benefits and outcomes of VMMC, awareness-raising campaigns on VMMC should also target women in fishing communities and other communities with high HIV prevalence, given their potential influence on male partners. Further studies are required to assess the implications of female partner support and involvement on VMMC uptake in the fishing communities.

Our study has some limitations. First, high mobility in fishing communities resulted in a non-response rate of 26.9% (many of these women were not found in their homes as they kept on moving to other fishing communities); this may have potentially reduced the power of our study. Second, due to the cross-sectional nature of this study, it was not possible to infer a causal relationship between women’s support for VMMC and the associated factors. However, the information generated can inform policymakers for appropriate interventions in these and similar communities. Third, the findings from the two fishing communities along the shores of Lake Victoria may have limited generalizability to other fishing communities in Uganda.

## Conclusion

Women’s support for VMMC was high among women in fishing communities along Lake Victoria in Uganda. However, women perceived they were not involved in VMMC programs for decision making, and most of them had several misconceptions and lacked adequate knowledge about the six-week abstinence period after circumcision. We recommend that the Ministry of Health and VMMC-implementing partners devise strategies to increase the involvement of women in VMMC decision-making. There is a need to effectively target female partners during VMMC awareness-raising interventions, to dispel their myths and misconceptions that may slow down service uptake. Future studies should assess the implications of female partner support and their involvement, in uptake of VMMC in the fishing communities.

## Data Availability

The datasets used and analyzed during this study are available from the corresponding author upon reasonable request.

## Abbreviations

aPR: Adjusted Prevalence Ratio
CI: Confidence Interval
cPR: Crude Prevalence Ratio
FGD: Focus Group Discussion
HIV: Human Immuno-deficiency Virus
MoH: Ministry of Health
STI: Sexually Transmitted Infection
VMMC: Voluntary Medical Male Circumcision
WHO: World Health Organization
